# SS‐31 does not prevent or reduce muscle atrophy 7 days after a 65 kdyne contusion spinal cord injury in young male mice

**DOI:** 10.14814/phy2.15266

**Published:** 2022-05-25

**Authors:** Zachary A. Graham, Jennifer J. DeBerry, Christopher P. Cardozo, Marcas M. Bamman

**Affiliations:** ^1^ Research Service Birmingham VA Medical Center Birmingham Alabama USA; ^2^ 9968 Department of Cell, Developmental, and Integrative Biology UAB Birmingham Alabama USA; ^3^ 9968 Department of Anesthesiology and Perioperative Medicine UAB Birmingham Alabama USA; ^4^ Center for the Medical Consequences of Spinal Cord Injury Bronx New York USA; ^5^ Medical Service James J. Peters VA Medical Center Bronx New York USA; ^6^ Icahn School of Medicine at Mount Sinai New York New York USA; ^7^ UAB Center for Exercise Medicine Birmingham Alabama USA; ^8^ Florida Institute for Human and Machine Cognition Pensacola Florida USA

**Keywords:** metabolomics, paralysis, respirometry, spinal cord injury, SS‐31

## Abstract

Spinal cord injury (SCI) leads to major reductions in function, independent living, and quality of life. Disuse and paralysis from SCI leads to rapid muscle atrophy, with chronic muscle loss likely playing a role in the development of the secondary metabolic disorders often seen in those with SCI. Muscle disuse is associated with mitochondrial dysfunction. Previous evidence has suggested targeting the mitochondria with the tetrapeptide SS‐31 is beneficial for muscle health in preclinical models that lead to mitochondrial dysfunction, such as cast immobilization or burn injury. We gave young male mice a sham (*n* = 8) or 65 kdyne thoracic contusion SCI with (*n* = 9) or without (*n* = 9) daily administration of 5.0 mg/kg SS‐31. Hindlimb muscle mass and muscle bundle respiration were measured at 7 days post‐SCI and molecular targets were investigated using immunoblotting, RT‐qPCR, and metabolomics. SS‐31 did not preserve body mass or hindlimb muscle mass 7 days post‐SCI. SS‐31 had no effect on soleus or plantaris muscle bundle respiration. SCI was associated with elevated levels of protein carbonylation, led to reduced protein expression of activated DRP1 and reductions in markers of mitochondrial fusion. SS‐31 administration did result in reduced total DRP1 expression, as well as greater expression of inhibited DRP1. Gene expression of proinflammatory cytokines and their receptors were largely stable across groups, although SS‐31 treatment led to greater mRNA expression of *IL1B*, *TNF*, and *TNFRSF12A*. In summation, SS‐31 was not an efficacious treatment acutely after a moderate thoracic contusion SCI in young male mice.

## Graphical abstract

SS‐31 is has been shown to be an efficacious treatment for many conditions of muscle dysfunction. We show that it does not protect losses in body or muscle mass after contusion SCI in young male mice.

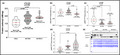

## 1 INTRODUCTION

Loss of muscle mass occurs rapidly after traumatic spinal cord injury (SCI). Immediate disuse during hospitalization and resulting paralysis from the anatomical injury are the major drivers of muscle atrophy during the acute days and weeks post‐SCI (Reid & Moylan, 2011). There are roughly 17,000 new incidences of SCI per year in the US and a total population of roughly 300,000 individuals with SCI in the US (National Spinal Cord Injury Statistical Center, 2021). Individuals with SCI face extensive direct and indirect financial costs from the injury, elevated risks of many cardiometabolic diseases when compared to able‐bodied individuals as well as a potential reduction in quality of life and independent living. Incomplete SCI has been the most common extent of injury in the past 5 y with falls and automobile accidents serving as the major causes of injury (National Spinal Cord Injury Statistical Center, 2021). Importantly, incomplete SCI allows a capacity for functional recovery in the weeks to months after the injury because once the cord is surgically or naturally decompressed around the lesion site, preserved axonal tracts begin to regain proper function across the lesion and the neuroplasticity program begins (Cote et al., 2017; Tran et al., 2018).

Skeletal muscle undergoes a well‐described process of rapid proteolytic breakdown when unloaded, followed by a sustained period of anabolic resistance (Bodine, 2013; Sartori et al., 2021). The muscle losses post‐SCI can be dramatic, with substantial losses observed within the first 6 weeks in humans (Castro et al., 1999; Castro et al., 1999) and further losses with an oxidative‐to‐glycolytic fiber‐type shift seen in those with long‐standing SCI (Moore et al., 2015; Yarar‐Fisher et al., 2013). In mice, muscle atrophy can be seen by 7 days after a severe contusion SCI (Bigford et al., 2018) and complete spinal cord transection (Graham et al., 2019). Mitochondrial derangements are typically seen alongside muscle atrophy though they are not well‐described after SCI. Magnetic resonance spectroscopy has shown in vivo oxidative function is reduced in those with SCI (Erickson et al., 2013; McCully et al., 2011). In pre‐clinical models, formoterol was able to rescue deficits in mitochondrial density and improve locomotor recovery (Scholpa et al., 2019, 2021). Thus, targeted interventions to improve mitochondrial function in paralyzed muscles may be an efficacious treatment. One promising intervention is the tetrapeptide SS‐31 (tradename Elamipretide). SS‐31 localizes to the inner membrane of the mitochondria (IMM) through surface charge density interactions with cardiolipin (Birk et al., 2013; Mitchell et al., 2020). It stabilizes membrane curvature and charge (Mitchell et al., 2020) and can improve mitochondrial protein function (Chavez et al., 2020). In additional pre‐clinical models, SS‐31 has prevented cast immobilization‐induced muscle atrophy and preserved markers of mitochondrial function (Min et al., 2011; Talbert et al., 2013).

We have previously shown that when compared to vehicle‐treated animals, SS‐31 was not able to prevent losses in gastrocnemius mass or body mass 14 days after a moderate contusion SCI in young male mice (Graham et al., 2021) That study had some limitations as the injury force and duration of post‐SCI recovery allowed for the return of loading and walking, potentially confounding those outcomes. Accordingly, the current study aimed to determine if daily treatment of SS‐31 could reduce the magnitude of body mass and muscle mass loss post‐acute SCI using a more severe injury force and shorter timeframe post‐injury, greatly reducing the potential of muscle loading post‐injury. Additional respiratory and molecular assays of hindlimb muscles were carried out to determine if SS‐31 may improve markers of mitochondrial function and muscle health post‐SCI.

## 2 MATERIALS AND METHODS

All materials used in this study can be found in Table S1 (https://www.doi.org/10.6084/m9.figshare.12925427).

### 2.1 Animals

8‐week old male mice were purchased from Charles River and acclimated for at least 1 week in a standard AAALAC‐accredited animal housing facility with a 12:12 light‐dark cycle and ad libitum access to chow and water. Male mice were specifically used for this study as males comprise 97% of the US Veteran SCI population (Curtin et al., 2012) as well as make up ~80% of all new SCI. (National Spinal Cord Injury Statistical Center, 2021) Additionally, approximately 24% of all injuries occur in young adults aged 17–22 and this age is roughly similar to the age of the mice used in this study (National Spinal Cord Injury Statistical Center 2020). 28 animals were initially used for the study but 1 SCI + Vehicle (SCI + VEH) animal and 1 SCI + SS‐31 animal were prematurely sacrificed due to complications from manual bladder expression. Thus, final group numbers were Sham (*n* = 8), SCI + VEH (*n* = 9), and SCI + SS‐31 (*n* = 9). The vehicle for this study was lactated Ringer’s solution. All studies were reviewed and approved by the Institutional Animal Care and Use Committee at the University of Alabama‐Birmingham (IACUC #: 21639).

### 2.2 Laminectomy and contusion SCI

Detailed methods for the laminectomy and thoracic contusion SCI have been described previously (Graham et al., 2021). Briefly 9–10‐week old mice were anesthetized with continuous respiration of 2%–5% isoflurane. The area over the spine was shaved, cleaned with 70% ethanol, then sterilized with betadine. An incision was made from T7‐T11 and the muscle along the spinal column was removed. The lateral processes of the vertebral arch of T9 and T10 were cut with sharp scissors and the vertebrae were carefully removed in one piece with fine forceps. Animals selected for a contusion SCI were placed within the clampable forceps of the Infinite Horizon Impactor and maintained under 2%–5% isoflurane. They received a 65 kdyne injury force with 0 s dwell time. 65 kdyne was chosen as it is severe enough to result in acute paralysis while also providing the capacity for some locomotor recovery (Bigford et al., 2018). Equal bilateral bruising of the spinal cord was confirmed before suturing and wound closure. Sham animals had the dura exposed following laminectomy before wound closure. All animals were placed in clean cages filled with Alpha‐Dri+bedding on warming pads and were singly‐housed for the rest of the study.

### 2.3 Post‐operative care and tissue harvest

Animals were administered carprofen (5.0 mg/kg) immediately and 24 h post‐surgery. Buprenorphine (0.1 mg/kg) was given immediately post‐surgery and then every 12 h for 3 days. We have previously shown that commercially‐prepared SS‐31 (GenScript) is bioactive in cell culture studies (Graham et al., 2021). The dose of SS‐31 (5.0 mg/kg) was chosen to match the highest dose published in studies focusing on skeletal muscle (Lee et al., 2011) as well as because 1.5 mg/kg was not sufficient to protect muscle mass 14 days after a sciatic nerve transection (Graham et al., 2021). Vehicle or SS‐31 was administered subcutaneously daily, which has been used in other pre‐clinical studies that aimed to improve hindlimb muscle health using SS‐31 (Min et al., 2011; Sakellariou et al., 2016; Talbert et al., 2013). All SCI animals had Basso Mouse Scale (BMS) locomotor scores ≤1 at the 24 h point post‐injury, providing evidence of severely diminished locomotor capacity. BMS was completed in an open‐field environment following the established procedures (Basso et al., 2006). Animals were given fruit crunch treats and standard chow, both placed on the cage floor throughout the entirety of the study to ensure access for paralyzed animals. Bladders were manually expressed 2–3 times a day.

Body mass was measured daily. Tissue was harvested from all animals 7 days post‐surgery while under continuous 2%–5% isoflurane anesthesia. All mice were between 10–11 weeks old at sacrifice. The soleus, plantaris, gastrocnemius, tibialis anterior (TA), and extensor digitorum longus (EDL) were carefully removed and weighed. The left soleus and plantaris were prepared for ex vivo respiration studies. All other muscles were flash‐frozen in dry‐ice cooled isopentane. Animals were euthanized by the combination of exsanguination and removal of the heart.

### 2.4 Serum assays

Blood was collected via ventricular puncture and serum was separated and aliquoted after a 20 min spin at 1500 g in a 4°C microcentrifuge. Serum levels of glucose (Invitrogen), insulin (Invitrogen), and triglycerides (Cayman Chemical) were determined using manufacturer’s guidelines. One animal from the SCI + VEH group did not have sufficient collected serum for analyses.

### 2.5 Ex vivo muscle oxygen consumption

The complete left soleus and 5–8 mg of the left plantaris of a subset of animals from each group (Sham *n* = 5, SCI + VEH *n* = 6, SCI + SS‐31 *n* = 7) were used for ex vivo high‐resolution oxygen consumption studies using an Oroboros O2k respirometer. After wet weights were recorded, the excised muscles were quickly placed in ice‐cooled B1 solution (250 mM sucrose, 10 mM Tris, 0.5 mM sodium EDTA and 1 g/l fatty acid free BSA) supplemented with protease and phosphatase inhibitors in an ice‐cooled petri dish, carefully teased apart with fine forceps to improve permeabilization, then transferred to a fresh 1.5 mL aliquot of B1 solution containing 30 µg/ml saponin and placed in an inverting rotisserie at 4°C for 30 min. Following the rotisserie step, muscles were placed in the chambers of the respirometer filled with hyperoxygenated (600–700 µM) Miro3 buffer (200 mM sucrose, 20 mM taurine, 20 mM HEPES, 10 mM KH_2_PO_4_, 3 mM MgCl_2_, 3 mM EGTA and 1 g/L fatty acid‐free BSA) warmed to 37°C. Muscle respiration was continuously recorded and substrates were added with Hamilton syringes. All concentrations listed are the final volume concentrations. The values were measured at baseline (State 1; no substrate) and after treatment with pyruvate and malate (State 2; 10 mM and 2 mM, respectively), ADP (State 3; 5 mM), oligomycin (State 4; 16 mg/ml), FCCP (uncoupled respiration; 2.5 µM) and antimycin A (State 5; 20 µM). All muscles were tested using the same respirometer and each muscle group was tested using the same chamber at the UAB Bio‐analytical Redox Biology Core. All respiration values were normalized to the tissue mass used for the experiment.

### 2.6 Tissue homogenization, protein expression, and carbonylation ELISA

Detailed procedures for tissue homogenization and protein expression have been reported (Graham et al., 2021). In brief, ~25 mg of the left gastrocnemius muscle was homogenized in RIPA buffer supplemented with protease and phosphatase inhibitors using a bead‐mill homogenizer. The homogenates rested on wet ice for 30 min then were spun at 14,000 g for 20 min at 4°C. The supernatant was collected and protein concentrations were determined using a microBCA kit. Protein expression and total protein were measured with the automated Wes 12–230 kDa capillary electrophoresis system (ProteinSimple) using 1.2 µg of protein per sample. Compass software (ProteinSimple) was used to quantify protein expression using the ‘High Dynamic Range’ image generated by the software with the ‘Drop Down’ algorithm. Studies that have validated the antibodies used in this study with knockout, knockdown or overexpression experiments are presented in Table S1. Of note, the OXPHOS antibody cocktail did not resolve in five distinct bands in our hands using the Wes system so its data was quantified by summing the band values from the 25–55 kDa range. Additionally, whole images of the blots can be found in File S1 (https://www.doi.org/10.6084/m9.figshare.12925439). Gastrocnemius homogenates were also used to determine protein carbonylation. 20 µg/ml of protein from the muscle homogenate was derivatized using dinitrophenylhydrazine. Carbonyl concentrations were determined by ELISA following the manufacturer’s instructions (Cell BioLabs).

### 2.7 RNA isolation and RT‐qPCR

RNA was isolated from ~25 mg of the left gastrocnemius using the miRNeasy kit according to the manufacturer’s guidelines (Qiagen). RNA concentrations were determined using a Nanodrop and 1 µg of RNA was used for reverse transcription. The resulting cDNA library was diluted 1:10 with nuclease‐free water and Taqman gene expression assays were used for all RT‐qPCR experiments. Comparisons of gene expression were completed using the 2^−ddCt^ method with the geometric mean of 18Sr and beta‐2‐microglobulin (B2 M) as the normalizing factor.

### 2.8 Mass spectroscopy

10 mg of the left gastrocnemius was sent to West Coast Metabolomics for untargeted metabolomics analyses using GC‐TOF mass spectroscopy. Data acquisition and processing were completed as reported (Fiehn et al., 2010). Peak spectra were normalized to the median, log transformed, and scaled using pareto scaling. Our group (Graham et al., 2019) and others (Aguer et al., 2017) have previously used these or similar methods to describe the metabolomics profile of mouse skeletal muscle. Targeted SS‐31 detection was completed using HILIC‐triple quadrapole mass spectroscopy as described (Hook et al., 2019) with 1.0 µg/ml of SS‐31 used as a reference standard.

### 2.9 Statistics

Absolute and normalized body mass differences were tested with two‐way mixed model ANOVAs, with a particular focus on differences among groups at each timepoint. One‐way ANOVAs with Tukey’s multiple comparisons were used for all other analyses. Data are represented by violin plots with median (solid red) and quartile (dotted black) lines. All plots are shown with effect size represented with *eta^2^* value, with general guidelines being *eta^2^* of 0.01 being a small effect, 0.06 being a moderate effect and 0.14 of being a large effect, as well as the ANOVA *p* value. We define a statistical threshold of meaningful differences at *p* < 0.10, although we acknowledge there are limitations with interpreting *p* values at arbitrary thresholds using null‐hypothesis testing (McShane et al., 2019). Group means and lower and upper bounds of 95% confidence intervals are shown in bold at the bottom of each violin plot. Between group brackets signify mean differences and are presented with the *p* value from Tukey’s follow‐up testing. Statistics were calculated using Prism 8.0 (GraphPad), with the exception of the metabolomics data, which was analyzed using MetaboAnalyst 4.0 (Chong et al., 2018). Every statistical comparison made for this study can be found in Table S2 (https://www.doi.org/10.6084/m9.figshare.12925478).

## 3 RESULTS

### 3.1 Body mass and Basso Mouse scale

**FIGURE 1:**
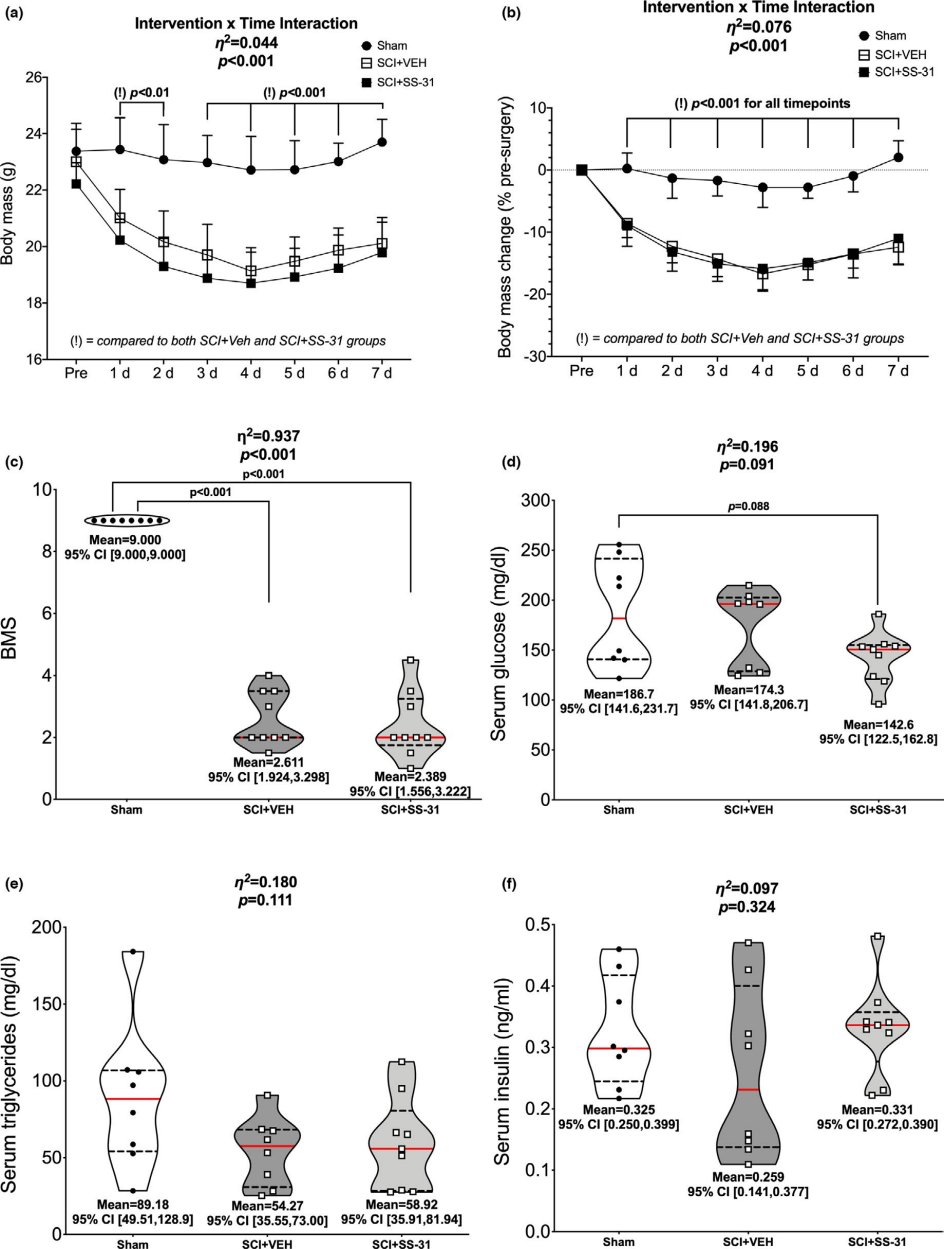
Body mass, BMS and circulating factors were measured after SCI. SCI led to reduced (a) absolute and (b) relative body mass changes across 7 days post‐contusion SCI but no effect of SS‐31. (c) BMS scores were greatly reduced after SCI, though no differences were observed between SCI groups after 7 days of SCI. (d) Serum glucose concentrations were reduced in the SCI+SS‐31 animals compared to sham animals but (e) serum triglycerides and (f) serum insulin were unchanged. Body mass data are presented as group means +SE for figure clarity. Non‐body mass data are represented by violin plots with median (solid red) and quartile (dotted black) lines. Group means and lower and upper bounds of 95% confidence intervals are shown in bold at the bottom of each violin plot. Between group brackets signify mean differences and are presented with the *p* value from Tukey’s follow‐up testing. Exact *p* values for every body mass comparison can be found in Table S2

There was a Group × Time interaction effect for absolute body mass (Figure 1a; *p* < 0.001; main effect of Group = *p *< 0.001, main effect of Time = *p *< 0.001), with losses observed in both SCI groups compared to Sham. Losses in mass compared to each SCI group’s respective pre‐value started 1 days post‐SCI and were sustained throughout the rest of the study. Similar outcomes were noted with relative body mass (Figure 1b; *p *< 0.001; main effect of Group = *p *< 0.001, main effect of Time = *p *< 0.001). BMS locomotor scores were reduced at 7 days post‐SCI in both vehicle and SS‐31‐treated animals compared to Sham (Figure 1c; *p *< 0.001).

### 3.2 Serum

There was a difference in serum glucose levels among groups (Figure 1d; *p *= 0.091) with the SCI + SS‐31 group having reduced concentrations compared to sham animals. There were no differences in serum triglyceride (Figure 1e; *p *= 0.111) or insulin levels (Figure 1f; *p *= 0.324) among the groups.

### 3.3 Tissue mass

**FIGURE 2:**
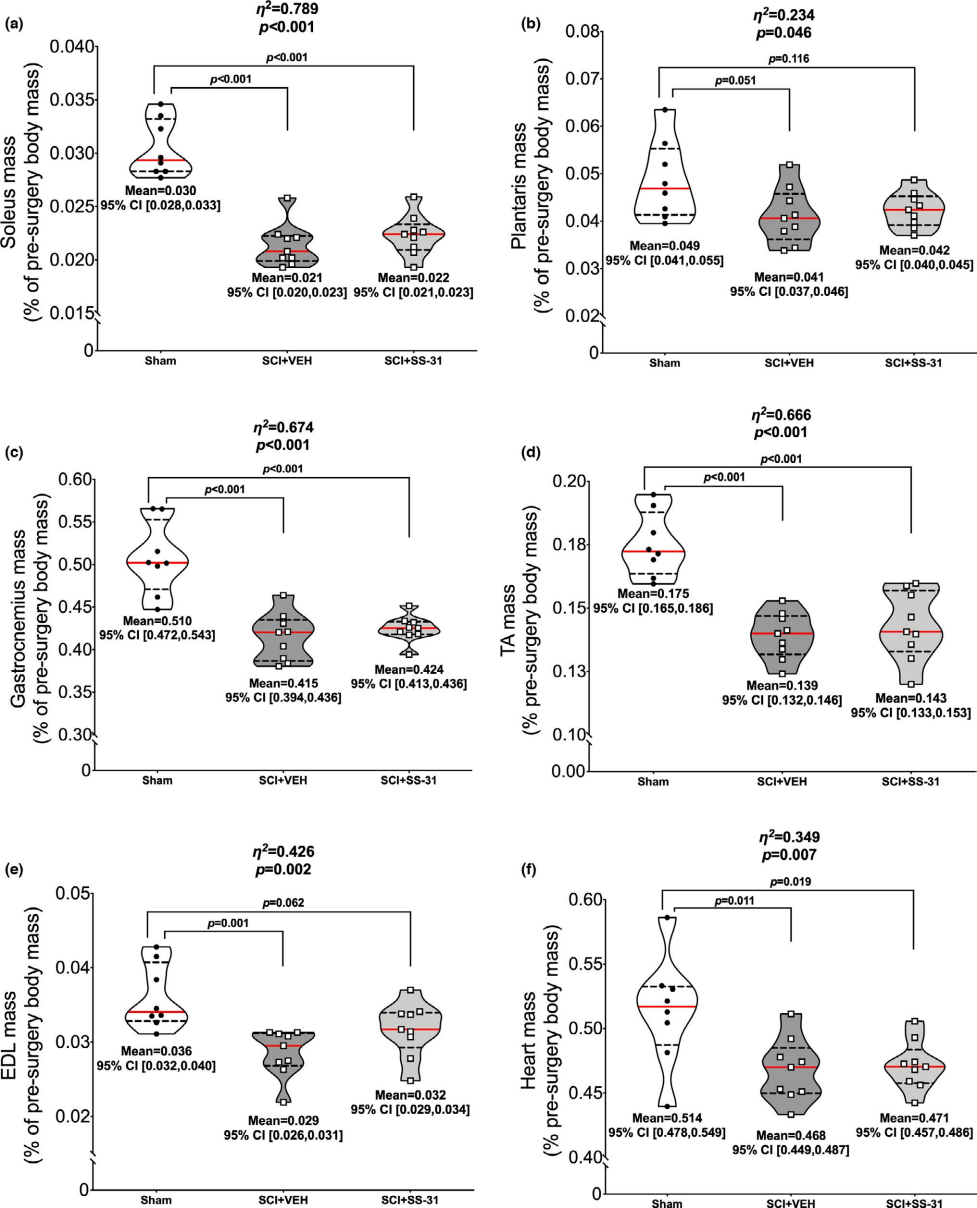
SS‐31 did not prevent losses in muscle mass. There was no effect of SS‐31 on muscle mass as both SCI groups had similar losses in normalized (a) soleus, (b) plantaris, (c) gastrocnemius, (d) TA, (e) EDL and (f) heart mass 7 days post‐contusion SCI when compared to sham animals. Data are presented with corresponding *eta* ^2^ and *p* values from ANOVA testing. All individual data points are shown in violin plots with median (solid red) and quartile (dotted black) lines. Group means and lower and upper bounds of 95% confidence intervals are shown in bold at the bottom of each violin plot. Between group brackets signify mean differences and are presented with the *p* value from Tukey’s follow‐up testing

There were reductions in normalized mass in both SCI groups compared to sham for the soleus (Figure 2a; *p *< 0.001), plantaris (Figure 2b; *p* = 0.046), gastrocnemius (Figure 2c; *p *< 0.001), TA (Figure 2d; *p *< 0.001), EDL (Figure 2e; *p *= 0.002) and heart (Figure 2f; *p *= 0.007).

### 3.4 High resolution oxygen consumption

**FIGURE 3:**
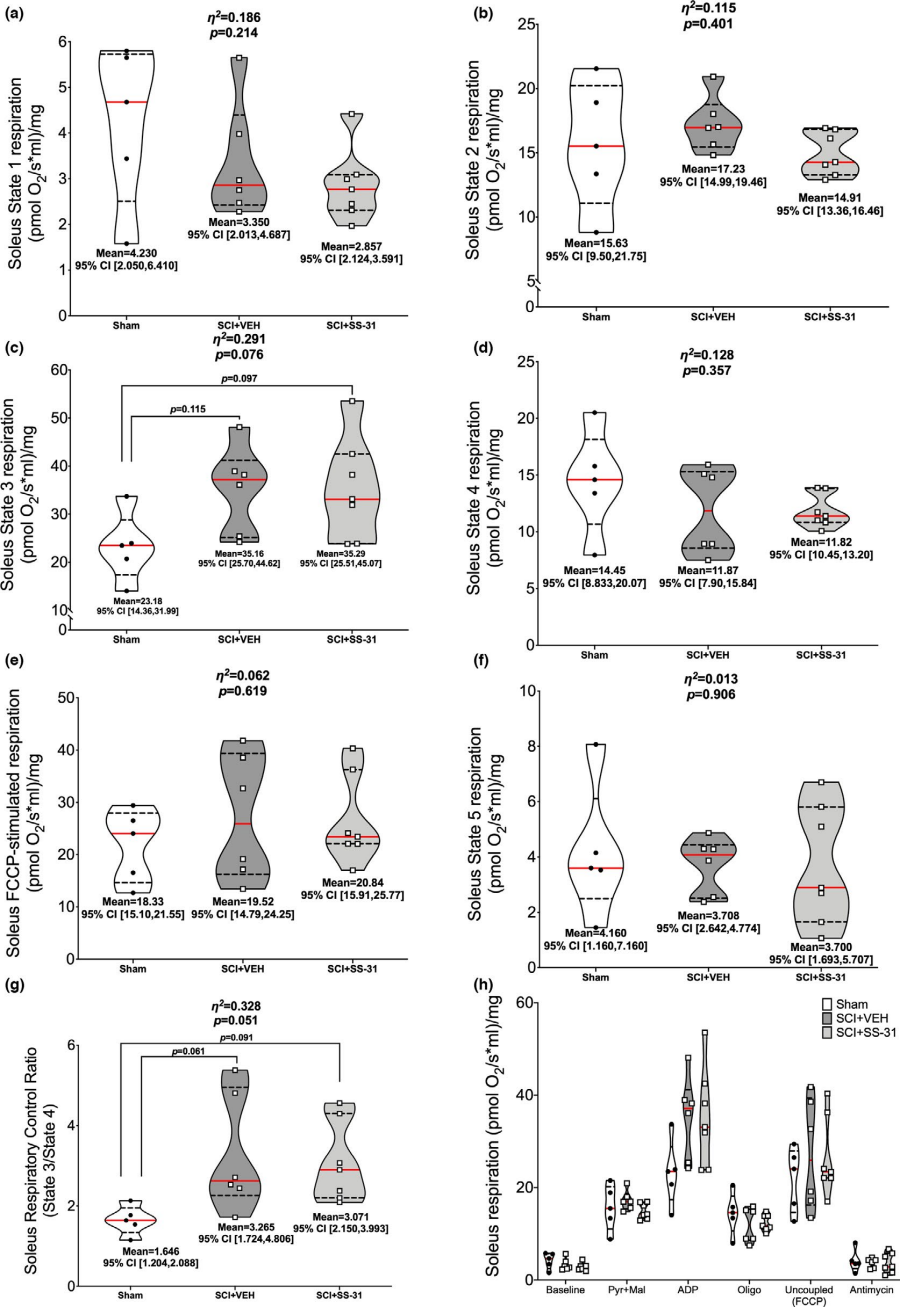
Ex vivo oxygen consumption of the soleus using high‐resolution respirometry. No changes were noted for (a) State 1 (baseline) or (b) pyruvate +malate‐stimulated State 2 respiration. Elevated oxygen consumption was seen in (c) ADP‐stimulated State 3 respiration but no further differences were observed for (d) oligomycin‐induced State 4 respiration, (e) FCCP‐induced uncoupled respiration, or (f) antimycin‐induced State 5 respiration. SCI led to elevated (g) respiratory control ratios (State 3/State 4). (h) Overall summary figure. Data are presented with corresponding *eta* ^2^ and *p* values from ANOVA testing. All individual data points are shown in violin plots with median (solid red) and quartile (dotted black) lines. Group means and lower and upper bounds of 95% confidence intervals are shown in bold at the bottom of each violin plot. Between group brackets signify mean differences and are presented with the *p* value from Tukey’s follow‐up testing

Figure 3 shows normalized soleus oxygen consumption rates. There was no difference among groups for State 1 (Figure 3a; *p *= 0.214) or State 2 (Figure 3b; *p *= 0.401) respiration. There were group differences in State 3 respiration (Figure 3c; *p *= 0.076) with increased oxygen consumption in both SCI‐groups compared to sham. There were no additional group differences in State 4 (Figure 3d; *p *= 0.357), uncoupled (Figure 3e; *p *= 0.619) or State 5 (Figure 3f; *p *= 0.906) respiration. There were group differences in RCR (Figure 3g; *p *= 0.051), with elevations observed in both SCI groups versus sham. Figure 3h shows all values in a summary plot.

**FIGURE 4:**
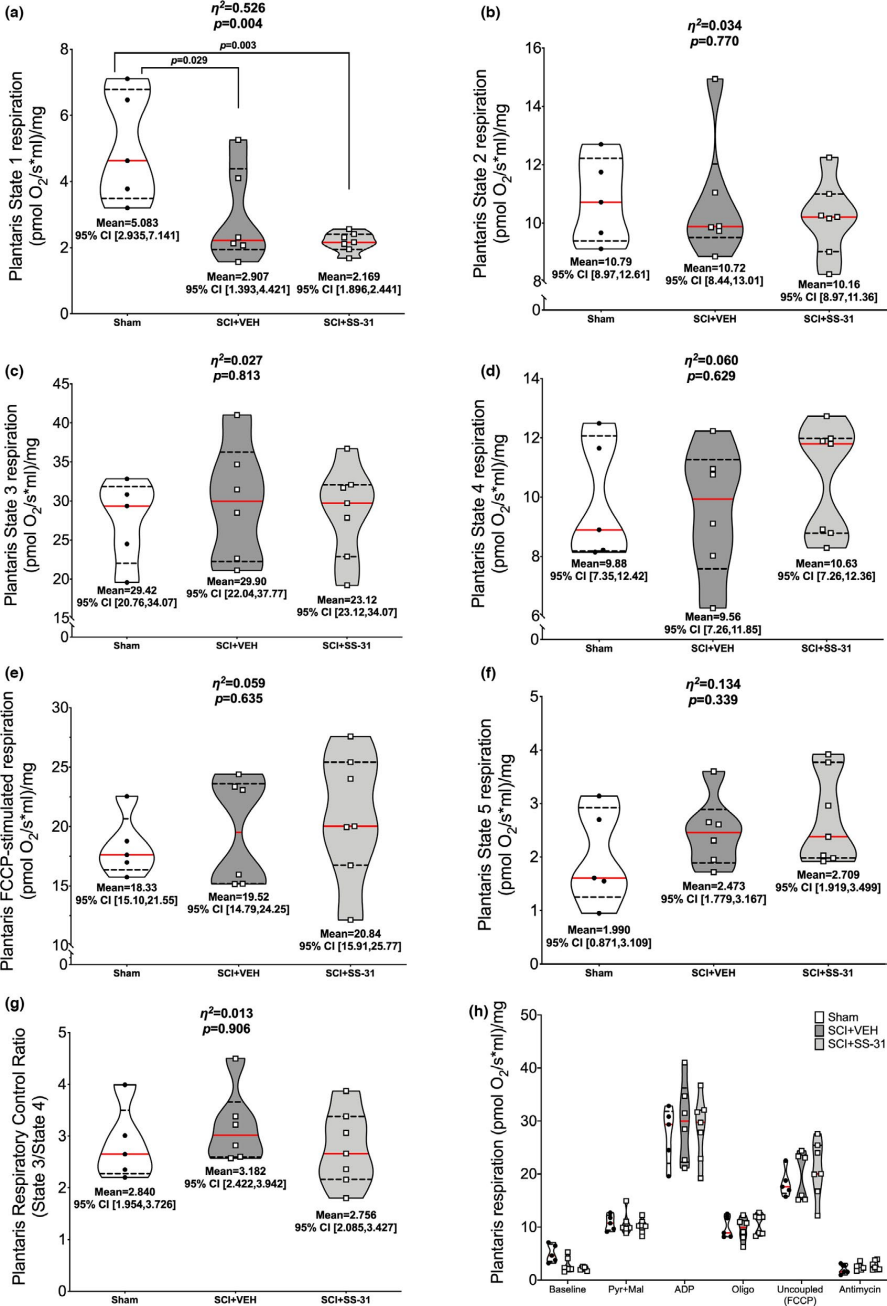
Ex vivo oxygen consumption of the plantaris using high‐resolution respirometry. SCI led to reduced (a) State 1 (baseline) respiration rates but no differences were observed during: (b) pyruvate +malate‐stimulated State 2 respiration, (c) ADP‐stimulated State 3 respiration, (d) oligomycin‐induced State 4 respiration, (e) FCCP‐induced uncoupled respiration or (f) antimycin‐induced State 5 respiration, or (g) respiratory control ratios (State 3/State 4). (h) Overall summary figure. Data are presented with corresponding *eta* ^2^ and *p* values from ANOVA testing. All individual data points are shown in violin plots with median (solid red) and quartile (dotted black) lines. Group means and lower and upper bounds of 95% confidence intervals are shown in bold at the bottom of each violin plot. Between group brackets signify mean differences and are presented with the *p* value from Tukey’s follow‐up testing

Normalized plantaris oxygen consumption rates can be seen in Figure 4. There were group differences in State 1 respiration (Figure 4a; *p *= 0.004), with reduced rates seen in the SCI groups compared to sham animals. No further group differences were noted for respiration during State 2 (Figure 4b; *p *= 0.770), State 3 (Figure 4c; *p *= 0.813), State 4 (Figure 4d; *p *= 0.629), uncoupled (Figure 4e; *p *= 0.635), State 5 (Figure 4f; *p *= 0.339) or RCR (Figure 4g; *p *= 0.906). Figure 4h shows all values in a summary plot.

### 3.5 Protein carbonylation

**FIGURE 5:**
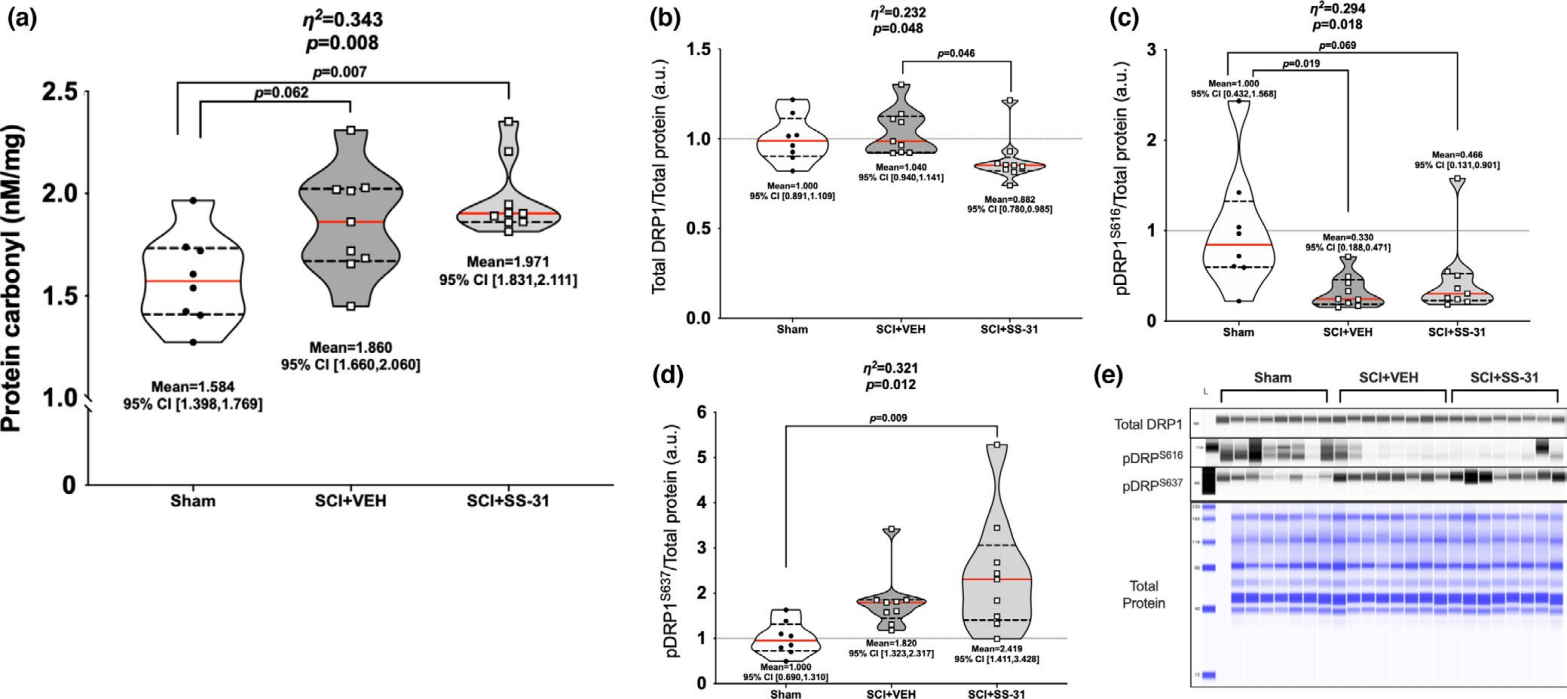
Changes it markers of gastrocnemius mitochondrial function. (a) SCI resulted in higher carbonyl concentrations in whole gastrocnemius lysates. Gastrocnemius protein expression of (b) total DRP1 was relatively stable, though SS‐31 resulted in reduced expression versus vehicle. (c) Marked reduction was observed after SCI for pDRP1^S616^, the main activation site, and elevations in (d) pDRP1^S637^, the main inhibitory site, in the SCI+SS‐31 animals compared to sham animals. (e) Representative images of each protein are shown in comparison to a total protein image. Data are presented with corresponding *eta* ^2^ and *p* values from ANOVA testing. All individual data points are shown in violin plots with median (solid red) and quartile (dotted black) lines. Group means and lower and upper bounds of 95% confidence intervals are shown in bold at the bottom of each violin plot. Between group brackets signify mean differences and are presented with the *p* value from Tukey’s follow‐up testing

There were group differences for protein carbonylation of gastrocnemius whole muscle lysate (Figure 5a; *p *= 0.008) with elevations in carbonyl concentration seen in both SCI groups versus sham.

### 3.6 Protein expression

**FIGURE 6:**
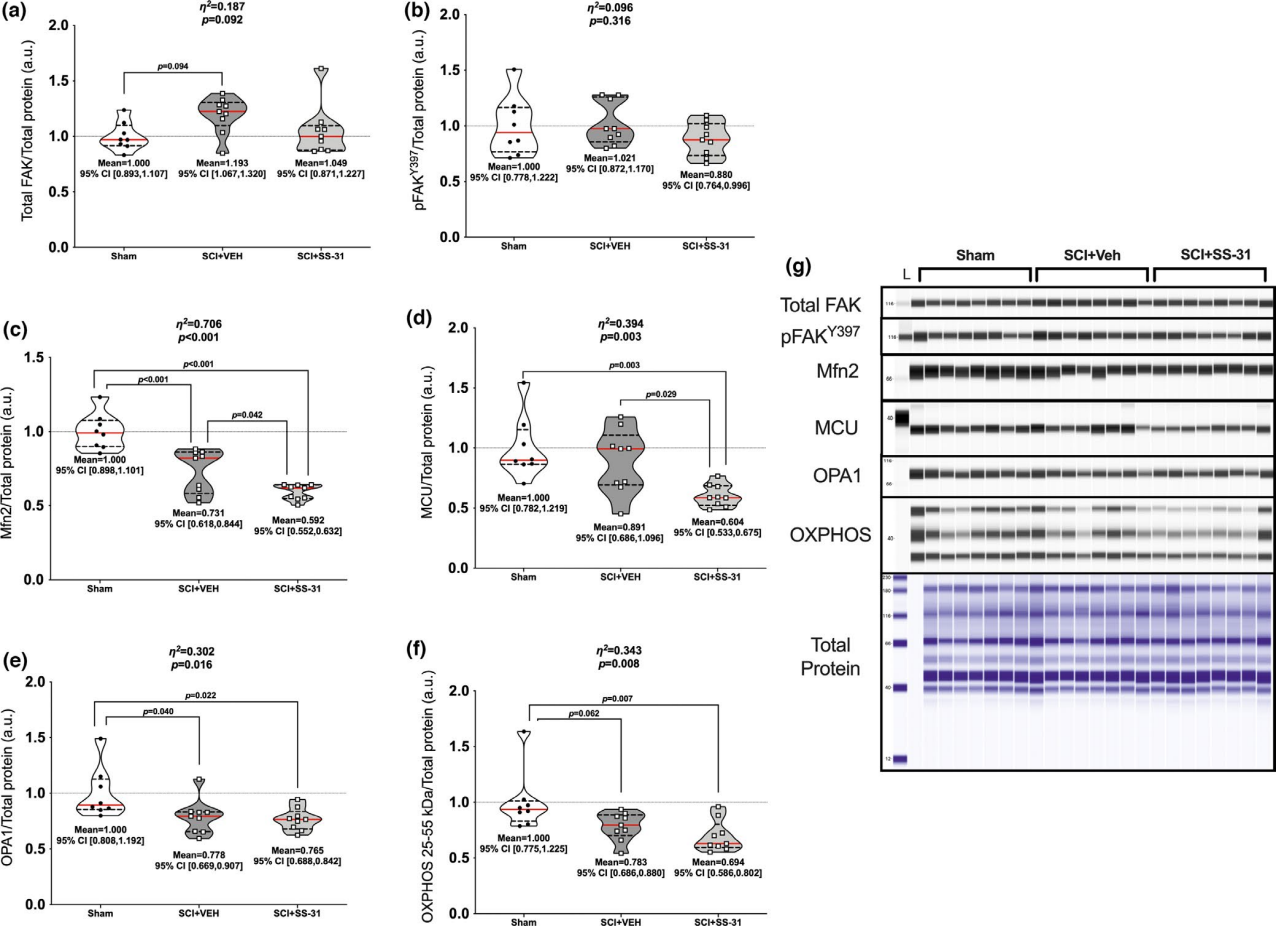
Changes in key fusion and mitochondrial inner‐membrane proteins of the gastrocnemius. The key mechanosensory FAK had (a) total expression levels elevated in the SCI‐VEH group compared to sham but no change in (b) its active site, pFAK^Y397^. Markers associated with mitochondrial fusion and membrane integrity, namely (c) Mfn2, (d) MCU, (e) OPA1 and (f) markers of the major electron transport chain subunits, were reduced after SCI, with SS‐31 being associated with further reductions in Mfn2 and MCU expression. (g) Immunoblots of each protein are shown in comparison to a total protein image. Data are presented with corresponding *eta* ^2^ and *p* values from ANOVA testing. All individual data points are shown in violin plots with median (solid red) and quartile (dotted black) lines. Group means and lower and upper bounds of 95% confidence intervals are shown in bold at the bottom of each violin plot. Between group brackets signify mean differences and are presented with the *p* value from Tukey’s follow‐up testing

Gastrocnemius muscle lysates were used to determine all protein expression levels. There were differences among groups for total DRP1 (Figure 5b; *p *= 0.048), with the SCI + SS‐31 groups having reduced levels compared to SCI+VEH. There were group differences in phosphorylation of DRP^S616^ (Figure 5c; *p *= 0.018), with both SCI groups being reduced compared to Sham. Phosphorylation of DRP^S637^ was different among groups (Figure 5d; *p *= 0.012), with the SCI + SS‐31 group being elevated compared to Sham animals. There were group differences in total FAK (Figure 6a; *p *= 0.092), with greater expression seen in the SCI + VEH animals compared to Sham, but no differences among groups for phosphorylated FAK^Y397^ (Figure 6b; *p *= 0.316). Mfn2 was expressed differently among groups (Figure 6c; *p *< 0.001) as expression was reduced in both SCI groups versus Sham, with further reductions seen in the SCI + SS‐31 group compared to the SCI + VEH group. The mitochondrial calcium uniporter (MCU) had differences among groups (Figure 6d; *p *= 0.003) as the SCI + SS‐31 group had reduced expression compared to Sham and SCI+VEH groups. OPA1 was differentially expressed among groups (Figure 6e; *p *= 0.016), as well as markers of subunits of the electron transport chain (Figure 6f; *p *= 0.008). Both of these factors were reduced for the SCI groups compared to Sham.

### 3.7 Gene expression

**FIGURE 7:**
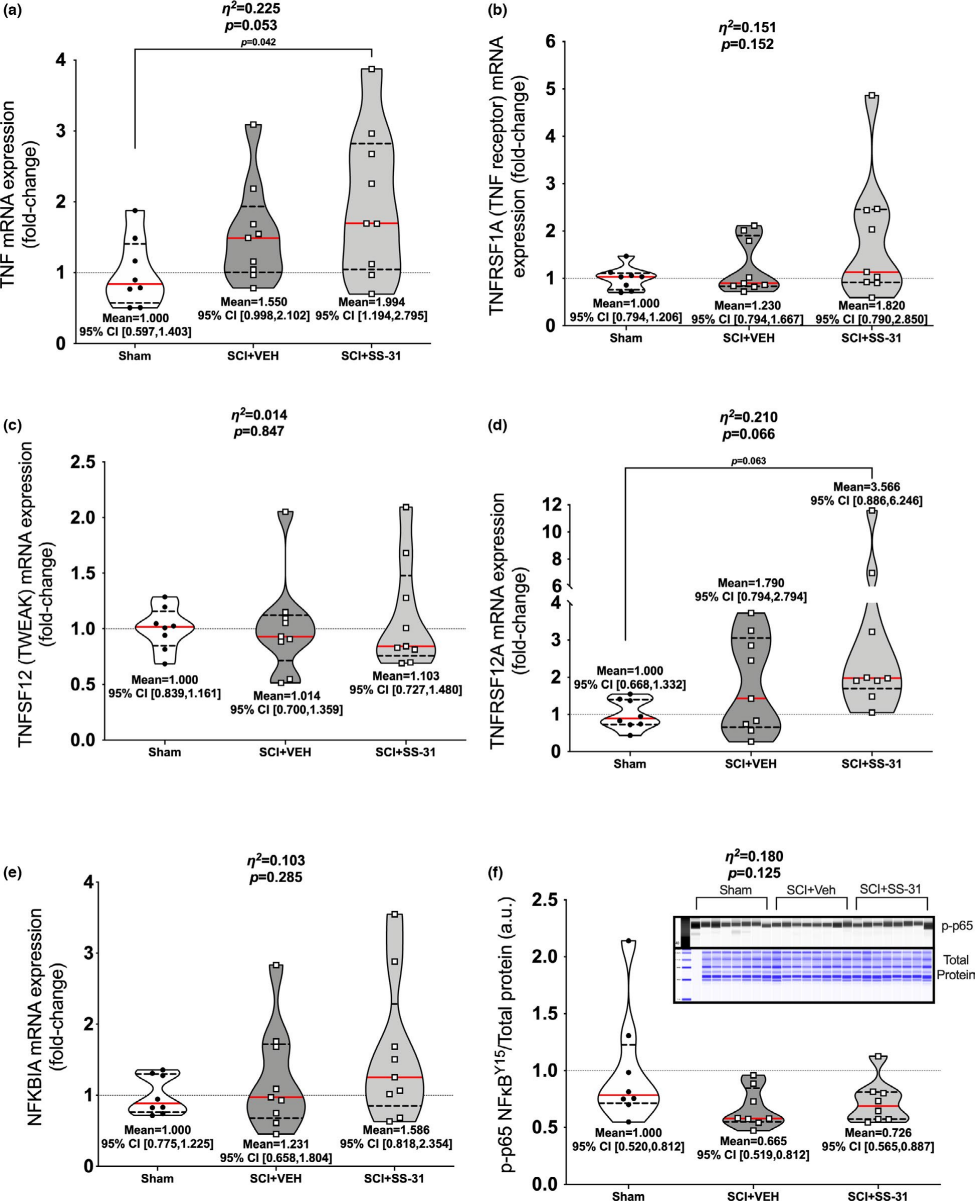
Gastrocnemius mRNA expression of the major cytokines associated with NFκB and muscle wasting. (a) *TNF* was upregulated in the SCI+SS‐31 group compared to Sham while no differences were noted for its receptor (b) *TNFRSF1A*. *(*c) *TNFSF12* was similar among groups but its receptor (d) *TNFRSF12A* was elevated in the SCI+SS‐31 group compared to Sham. (e) *NFKBIA* was unchanged among groups and (f) protein expression p‐p65^Y15^, a marker of protein activation, was not similar to the gene expression patterns of upstream activators. Data are presented with corresponding *eta* ^2^ and *p* values from ANOVA testing. All individual data points are shown in violin plots with median (solid red) and quartile (dotted black) lines. Group means and lower and upper bounds of 95% confidence intervals are shown in bold at the bottom of each violin plot. Between group brackets signify mean differences and are presented with the *p* value from Tukey’s follow‐up testing

**FIGURE 8:**
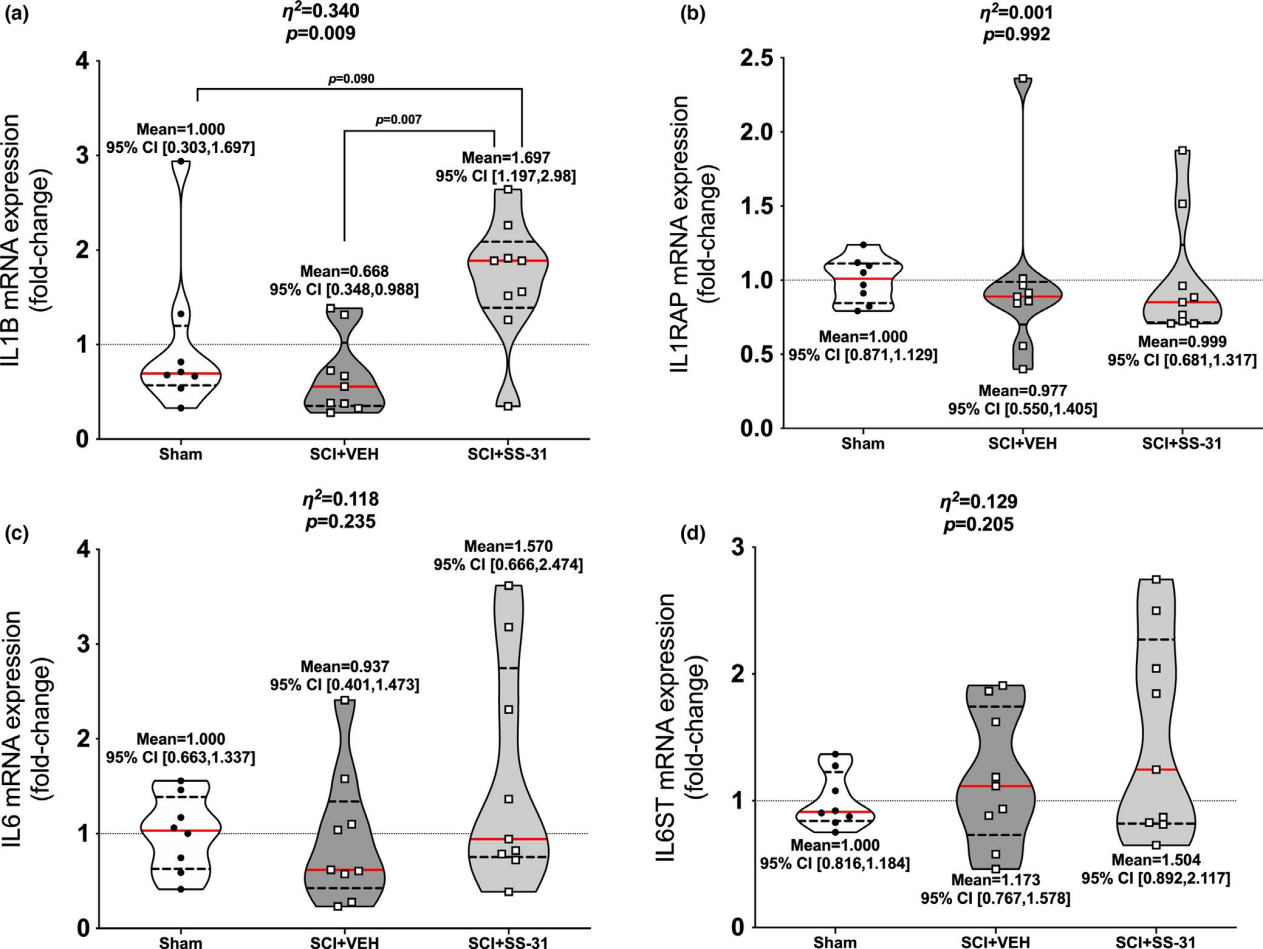
Gastrocnemius gene expression of key interleukins associated with muscle atrophy. (a) *IL1B* is up‐regulated in SCI+SS‐31 animals compared to Sham and SCI+VEH. No changes were seen with its receptor (b) *IL1RAP* as well as *(*c) *IL6* and its main effector receptor (d) *IL6ST*. Data are presented with corresponding *eta* ^2^ and *p* values from ANOVA testing. All individual data points are shown in violin plots with median (solid red) and quartile (dotted black) lines. Group means and lower and upper bounds of 95% confidence intervals are shown in bold at the bottom of each violin plot. Between group brackets signify mean differences and are presented with the *p* value from Tukey’s follow‐up testing

Gastrocnemius muscle was used for all gene expression assays. *TNF* was altered among groups (Figure 7a; *p*=0.053), with elevations in the SCI + SS‐31 group compared to Sham. The expression of its receptor, *TNFRSF1A*, was not changed (Figure 7b; *p *= 0.152). There were no differences among groups for *TNFSF12* [commonly known as TWEAK (Figure 7c; *p *= 0.847)] but there was an altered expression of its receptor *TNFRSF12A* [Fn14; Figure 7d; *p *= 0.066) as the SCI + SS‐31 group was elevated compared to Sham. There were no differences among groups for *NFKBIA* (Figure 7e; *p *= 0.285) or in protein phosphorylation of the major activation site for p65 NFκB (Figure 7f; *p *= 0.125). mRNA expression of *IL1B* was different among groups (Figure 8a; *p *= 0.009) with greater expression in the SCI + SS‐31 group compared to both Sham and SCI + VEH groups. There were no differences among groups for the main IL‐1β receptor effector *IL1RAP* (Figure 8b; *p *= 0.992). Expression of *IL6* mRNA (Figure 8c; *p *= 0.2349) or that of its main receptor effector *IL6ST* GP130 (Figure 8d; *p *= 0.205) were not altered among groups. Gene expression for markers of oxidative muscle and E3 ligases are shown in Figure S2 (https://www.doi.org/10.6084/m9.figshare.13110530). There were no differences among groups for *PPARG1A* (Figure S2a; *p* = 0.443), *MYH7* (Figure S2b; *p* = 0.199) or *PTK2* (Figure S2c; *p* = 0.272). Figure S3 can be found at: (https://www.doi.org/10.6084/m9.figshare.14527089). There were no changes among groups for the major muscle E3 ligases *FBOX32* (*p* = 0.285) or *TRIM63* (*p* = 0.257), or the mitochondrial‐focused E3 ligases *MUL1* (*p* = 0.484) or *PARK2* (*p* = 0.298).

### 3.8 Metabolomics

**FIGURE 9:**
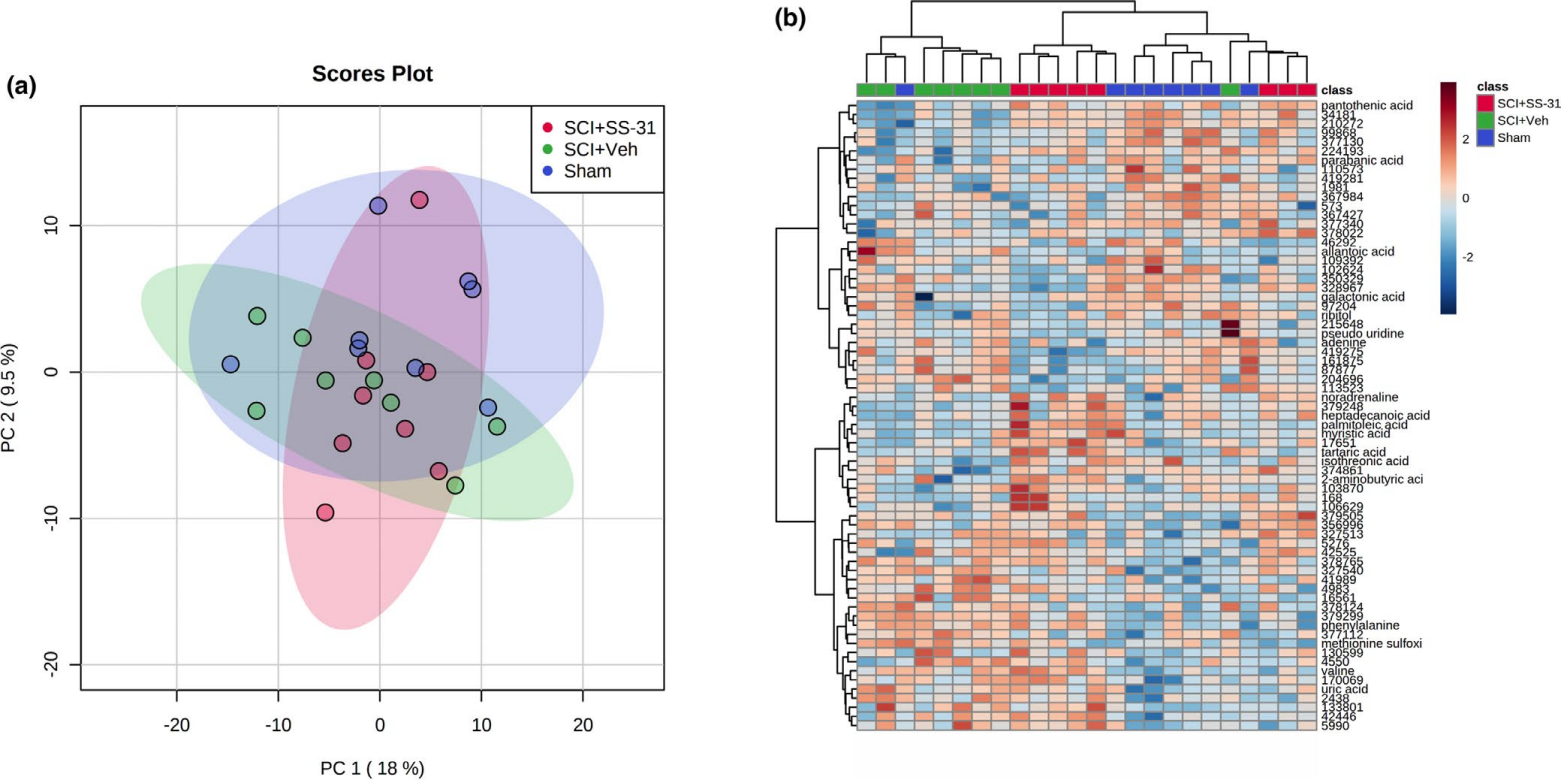
Untargeted primary metabolomics of whole gastrocnemius muscle was not able to identify key patterns among groups 7 days post‐SCI. (a) PCA shows no unique clustering of groups. (b) A hierarchical heatmap of the metabolites with raw *p* values of <0.10 shows some consistent grouping of the SCI+VEH and Sham groups but no clear patterns

Five hundred and three distinct spectra were detected from whole gastrocnemius muscle with 159 of them matching confirmed metabolites. Unsupervised principal components analysis of the 503 distinct spectra shows no clear separation of groups (Figure 9a). Sixty nine metabolites had a raw *p* < 0.10, though none met an FDR threshold of *p* < 0.10 (Table S3; https://www.doi.org/10.6084/m9.figshare.12925565). A hierarchical clustering heatmap of these 69 metabolites does not show distinct metabolomic patterns among groups (Figure 9b). A priori comparisons of the SCI + VEH and SCI + SS‐31 groups resulted in 58 metabolites with a *t*‐test comparison of *p *< 0.10, though none had an FDR of *p* < 0.10. (Table S5; https://www.doi.org/10.6084/m9.figshare.12925568). Raw data is provided in Table S6 (https://www.doi.org/10.6084/m9.figshare.12925574, Figure 9).

## 4 DISCUSSION

The ability of muscle mitochondria to quickly lose functional capacity in response to disuse leads to or sustains muscle atrophy (Hyatt et al., 2019). Acute SCI can pose unique challenges to skeletal muscle. Beyond the resultant disuse and paresis/paralysis from the anatomical injury, muscle is susceptible to the major metabolic stresses from the direct trauma that injured the spinal cord (e.g,. car accident), followed by any necessary surgical procedure (O’Shea et al., 2017). These factors can be accompanied by nutritional deficits due to losses in appetite and dysphagia that often accompany SCI (Thibault‐Halman et al., 2011). SS‐31 has shown efficacy in protecting muscle health and metabolic function in many pre‐clinical models that lead to muscle wasting and reduced muscle health (Campbell et al., 2019; Lee et al., 2011; Min et al., 2011; Montalvo et al., 2020; Powers et al., 2011; Righi et al., 2013; Sakellariou et al., 2016; Talbert et al., 2013). Despite the clear line of evidence showing SS‐31 is effective in preserving muscle health, our data suggests daily administration of 5.0 mg/kg does not provide protection for skeletal muscle in the acute phase of a moderate‐severe contusion SCI in mice.

SS‐31 showed no protective effect on SCI‐induced losses in body mass or hindlimb muscle mass. This was similar to our previous study that used a lower contusion force and longer recovery time (Graham et al., 2021). SS‐31 was able to preserve the mass of the soleus and plantaris when cast‐immobilized for 7 days in rats (Talbert et al., 2013) and after 14 d in mice (Min et al., 2011). The reasons for this discrepancy are not clear but systemic metabolic stress and reduced circulation may be factors. Losses in body mass are consistently seen in the acute stages of SCI, both in clinical and pre‐clinical settings. The manifestations are from obligatory muscle loss and transient trends in losses in body fat percentage despite reduced resting energy expenditure (Felleiter et al., 2017). In pre‐clinical settings, there is little doubt that reduced food intake post‐SCI likely exacerbates, at least temporarily, the major loss in body mass from the surgical intervention and traumatic injury itself as food intake is reduced across 28 days in rats with light‐moderate contusion SCI (Harris et al., 2019). Our published studies with longer periods of follow‐up after a complete spinal cord transection show mice start to regain weight 7–14 days post‐SCI (Graham et al., 2015; Graham, Liu, et al., 2020), and can return to baseline levels around 12 weeks after injury despite severe hindlimb atrophy and losses of 40%–75% of the mass of major fat depots (Graham, Liu, et al., 2020). Hydration is usually not a major issue in the acute stages of pre‐clinical SCI in our hands as mice get routine supplemental lactated Ringers and additional volumes needed for drug administration, both experimental and analgesic. As for skeletal muscle, unique changes as a result of SCI, such as reductions in tonic motor neuron signaling, potential denervation, reduced membrane potential and increased sarcolemmal permeability (Cisterna et al., 2020; Grumbles & Thomas, 2017) may alter SS‐31 kinetics and disrupt its localization to the IMM of skeletal muscle. We did not detect SS‐31 in the gastrocnemius muscles (Table S6; https://www.doi.org/10.6084/m9.figshare.14384495) and we did not use any localization assays like conjugated fluorescence markers so it is not possible to confirm drug delivery. SS‐31 peaks in concentration 2 h post‐injection in highly metabolic tissue like the liver and heart but rapidly decreases by 16 h post‐injection (Birk et al., 2013). The animals used in this study were sacrificed roughly 24 h after their last injection so it is not surprising there was a lack of confident SS‐31 detection.

It was previously thought the dimethyltyrosine of SS‐31 acts as a ROS scavenger but interactions with cardiolipin seem to be SS‐31’s principal mechanism of action (Birk et al., 2013; Mitchell et al., 2020). Cross‐linking mass spectrometry also shows SS‐31 directly interacting with proteins of the IMM (Chavez et al., 2020). We used protein carbonylation as a marker of oxidative stress using whole muscle gastrocnemius lysates and demonstrate SCI leads to elevated protein carbonyl concentrations with no effect of SS‐31. This is not in agreement with other disuse studies, along with evidence in aged skeletal muscle, which show SS‐31 reducing H_2_O_2_ release and other markers of oxidative stress (Min et al., 2011; Sakellariou et al., 2016; Talbert et al., 2013). Additionally, we show SS‐31 had no effect on the rate of oxygen consumption in permeabilized soleus or plantaris muscles after SCI. Hindlimb muscle disuse reduces mitochondrial function by 7 days, with some parameters affected as soon as 3 days post‐unloading (Trevino et al., 2019). While we did not note major differences in many of the respiratory states among groups for the soleus and plantaris muscles, there was an SCI‐induced reduction in baseline plantaris oxygen consumption. However, the increase in RCR in the soleus in the SCI groups was the inverse of what has been previously reported for hindlimb cast immobilization or hindlimb unloading (Kwon et al., 2016; Min et al., 2011; Talbert et al., 2013; Trevino et al., 2019). This increase in RCR was driven by ADP‐stimulated State 3 respiration and suggests increased ADP sensitivity. A cervical‐level SCI was associated with mean elevations in State 3 and State 4 mitochondrial respiration 24–36 h post‐SCI in the rat diaphragm (Smuder et al., 2016), though the oxidative and muscle fiber phenotype of the rat diaphragm and mouse soleus are not identical. Despite this, overall RCR was reduced compared to control animals (Smuder et al., 2016, 2020). Factors like creatine levels, adenine nucleotide transporter post‐translational modifications, and diet can affect ADP sensitivity and lead to changes in skeletal muscle mitochondrial respiration (Holloway, 2017). Our gastrocnemius metabolomics data did not provide any clear insight into whether ADP, creatine or other important metabolite associated with ADP sensitivity, though some caution is warranted in making comparisons across muscle groups. It is not clear why a moderate‐severe thoracic contusion SCI would lead to such differences in ADP‐stimulated respiration but one explanation could be difficulties with thermoregulation. Adult rats with a T4 transection had a 1°C reduction in core body temperature 7 days post‐SCI (Laird et al., 2006) and mice with a T3 transection show an inability to maintain body heat when challenged with a short‐term cold stress (Järve et al., 2018). Mice with an identical, thoracic‐level 65 kdyne contusion SCI have ~10% reduced 24 h energy expenditure 14 days after injury (Bigford et al., 2021) and, in rats, reduced cage activity, reduced caloric intake, but an increase in oxygen consumption, have been observed acutely post‐SCI (Harris et al., 2019). Thus, it is plausible, though certainly speculative, that muscle is trying to create heat in a non‐shivering manner through mechanisms involving the sarcoplasmic reticulum (Periasamy et al., 2017). Future pre‐clinical studies tightly controlled for and designed around isothermal, isocaloric, and indirect calorimetry would be incredibly beneficial for the field.

We also report extensive down‐regulation in phosphorylation of DRP1^S616^ post‐SCI. DRP1 is the principal mitochondrial fission protein and phosphorylation of DRP1^S637^ inhibits its action and sequesters it in the cytosol (Otera et al., 2013). It remains there until DRP1^S616^ phosphorylation, after which it is recruited to the OMM (Loson et al., 2013). Activated DRP1 oligomerizes at constriction points with the sarco/endoplasmic reticulum to initiate mechanical fission of the mitochondria (Kraus & Ryan, 2017; Mears et al., 2011). We have previously noted a sciatic nerve transection does not change gastrocnemius levels of total and activated DRP1 at 7 days post‐paralysis (Graham et al., 2018). Other studies have shown no change or trends for reduced total DRP1 protein expression 3 and 7 days after hindlimb unloading (Cannavino et al., 2015) as well as 7 (Iqbal et al., 2013) and 10 days (Tamura et al., 2015) post‐sciatic nerve transection. This reduction in activated DRP1 was not associated with protections in protein expression of Mfn2, OPA1 and components of the electron transport chain, as these key mitochondrial factors were similarly reduced in SCI animals. While these factors were sharply reduced post‐SCI, mRNA expression of the key mitochondrial biogenesis regulator *PPARGC1A*, had only slight mean reductions in expression after injury, suggesting another potential mechanism that may result in reductions in protein expression. Lastly, we found SS‐31 was associated with clear reductions in protein expression of MCU, an integral IMM calcium channel (Mammucari et al., 2016). This effect has no clear explanation but it may have to do with SS‐31 preferentially binding to cardiolipin in the IMM (Birk et al., 2013; Mitchell et al., 2020), an outcome which may alter the electrostatic boundary layer of the IMM and alter how calcium interacts with IMM proteins (Mitchell et al., 2020).

We have previously demonstrated that SCI does not result in major changes in the expression of pro‐inflammatory cytokines 14 days after SCI in pre‐clinical models of contusion SCI (Graham et al., 2021; Graham, Goldberger, et al., 2020) but in this report, we show gene expression assays of cytokines associated with muscle atrophy show an interesting pattern of upregulation in SCI + SS‐31 animals. Elevations in *IL1B* in the SCI + SS‐31 group compared to sham, as well as elevations in mRNA of factors upstream of NF‐κB signaling, namely *TNF* and the TWEAK receptor, *TNFRSF12A*, suggest a state of mild inflammation. Changes in these factors are interesting as SS‐31 reduced the release of TNFα from C2C12 myotubes as well as prevented TNFα‐induced release of other inflammatory markers (Lightfoot et al., 2015). These upregulations were not associated with changes in activated p65‐NF‐κB and they may not be having an appreciable effect on muscle signaling. Additional gene expression assays for type I oxidative markers, like PGC1α, FAK, and slow myosin, were not largely different among groups. Key E3 ligases associated with skeletal muscle breakdown, namely *FBOX32* and *TRIM63*, had large mean changes post‐SCI, but these were driven by a few mice with high‐expression levels. mRNA expression of *PARK2* and *MUL1*, E3 ligases related to the mitochondria were stable after SCI. These two molecules are reduced with PGC1α overexpression (Kang & Ji, 2016) and with the limited differences among groups we note for *PPARGC1A*, the gene encoding PGC1α, it would be expected that no major changes to these ligases would occur.

There are some clear limitations of our study. Perhaps the most important limitation was the use of a commercially‐prepared version of the drug. SS‐31 is a relatively simple tetrapeptide and we have shown our commercially‐prepared version is able to rescue oxygen consumption in cell culture studies. Although SCI is a unique experimental model with many systemic effects in the acute timespan post‐injury that may limit the effectiveness of SS‐31, commercially‐prepared SS‐31 has been demonstrated to be effective in limiting lung damage and reducing pulmonary inflammation during acute SCI in mice (Zhu et al., 2017). The QA/QC report generated by the commercial provider, which includes peptide spectra and molecular weight determined by mass spectroscopy as well as solubility profiles, are identical to what would be expected from SS‐31. So while we have no direct evidence of successful uptake, the general tissue pharmokinetics established by the Szeto group (Birk et al., 2013; Szeto, 2014), the aforementioned literature, our previous publication showing commercially‐prepared SS‐31 as bioactive in C2C12 myoblasts (Graham et al., 2021), and data within our current report (group differences for MCU protein expression and gene expression assays) provide strong evidence for uptake. However, we cannot exclude potential in vivo differences in the commercially‐prepared compound used in our study compared to the lead molecule. Another limitation is the use of a contusion SCI, while more clinically relevant, leads to altered rates of recovery between animals despite tight controlling of the surgical area, impact force, and the necessary requirement of having a 24 h post‐SCI BMS score of ≤1 (slight or no ankle movement). The differences in range of recovery would be expected to provide some variability in outcomes. For example, using a complete transection model we have shown clear group clustering by PCA and changes in metabolomics profiles 7 days post‐injury, with major differences in levels of glucose metabolites (Graham et al., 2019). In this current report the SCI groups show some orthogonal clustering along with the two major principal components but the majority of clustering is shared with sham animals and no metabolite passed the FDR threshold. We did not quantify lesion volume or analyze aspects of the spinal cord, so uniformity of injury cannot be confirmed, though all animals used in this study showed similar locomotor scores 24 h post‐SCI, demonstrating the consistency of initial injury. Our study only utilized young adult male mice to maintain consistency with our previous study, and it is acknowledged our results may not be reflective of studies using more mature young adults, as well as older and/or female mice. We cannot confirm sufficient delivery of SS‐31. SS‐31 was not reliably detected in the skeletal muscle of animals treated with the drug using a targeted mass spec approach, though aspects of our study, such as the distinct down‐regulation of MCU in the SCI+SS‐31 group, provide strong evidence the drug was being delivered. Our use of subcutaneous SS‐31 injections follows previous literature, (Min et al., 2011; Sakellariou et al., 2016; Talbert et al., 2013) but it is possible intraperitoneal injections or osmotic pumps may be better delivery systems. We did not control for food intake or monitor feeding so we cannot rule out that some of our data was affected by nutritional deficits.

## 5 CONCLUSION

SS‐31 was not able to attenuate or prevent SCI‐induced losses in body or muscle mass and is likely not an efficacious intervention to protect muscle health and function post‐acute SCI in young male mice.

## CONFLICT OF INTERESTS

The authors have no conflicts of interest to disclose.

## AUTHOR CONTRIBUTIONS

Research concept and study design: Zachary A. Graham and Christopher P. Cardozo; data collection: Zachary A. Graham; statistics and data interpretation: Zachary A. Graham; provided resources, reagents and equipment: Zachary A. Graham, Jennifer J. DeBerry, Christopher P. Cardozo, Marcas M. Bamman; preparation of manuscript: Zachary A. Graham; reviewing and editing manuscript: Zachary A. Graham, Jennifer J. DeBerry, Christopher P. Cardozo, Marcas M. Bamman.

## ETHICS STATEMENT

All studies were reviewed and approved by the Institutional Animal Care and Use Committee at the University of Alabama‐Birmingham.

## Supporting information



Fig S1

Fig S2

Table S1

Table S2

Table S3

Table S4

Table S5

Table S6
