# Supplementary figures and images for: SS‐31 does not prevent or reduce muscle atrophy 7 days after a 65 kdyne contusion spinal cord injury in young male mice
Source: Physiol Rep. 2022 May 25;10(10):e15266. doi: 10.14814/phy2.15266 (PMC9131615; doi:10.14814/phy2.15266)

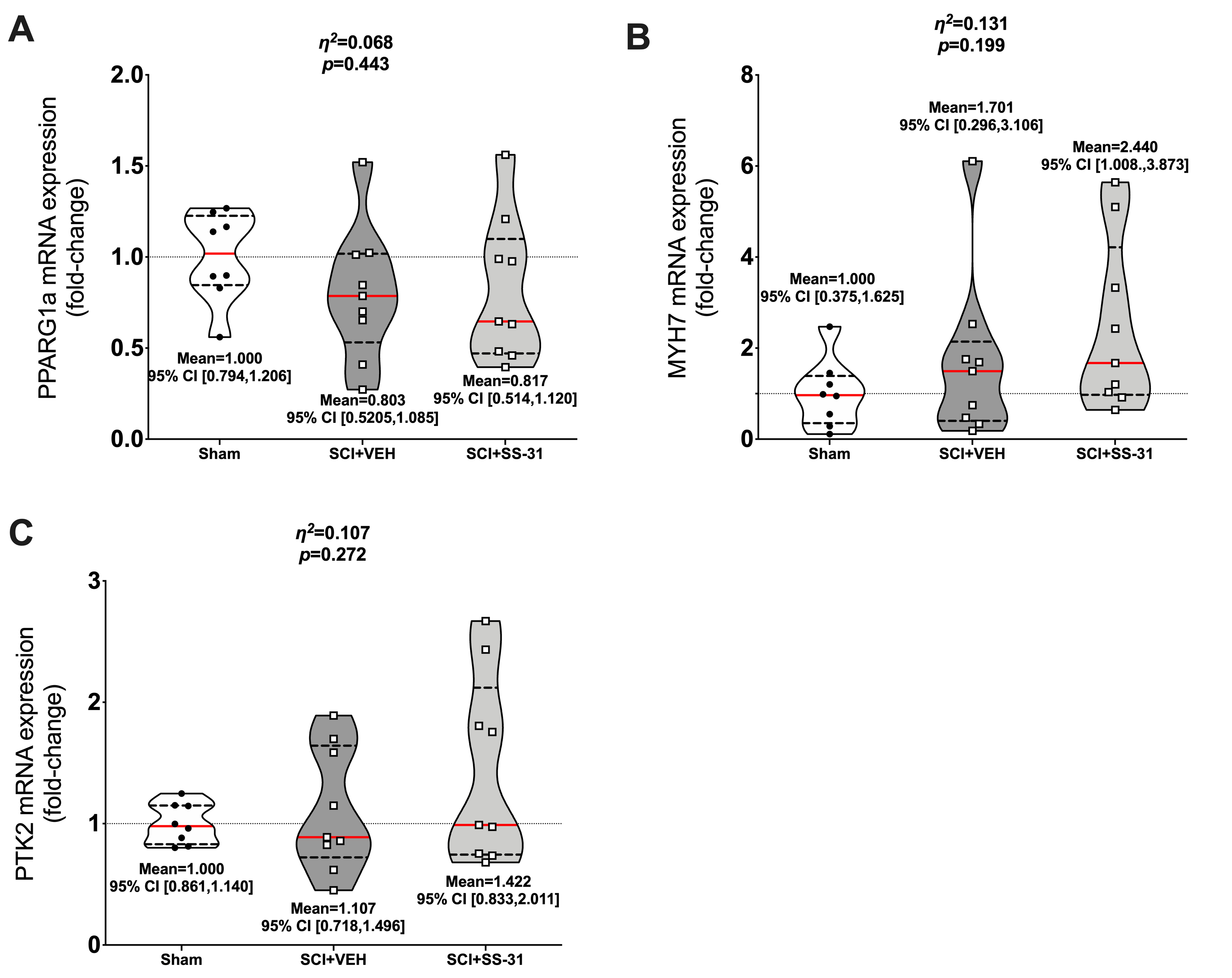

Supplement: Supplementary file 1 — Fig S1 [file PHY2-10-e15266-s008.tiff]

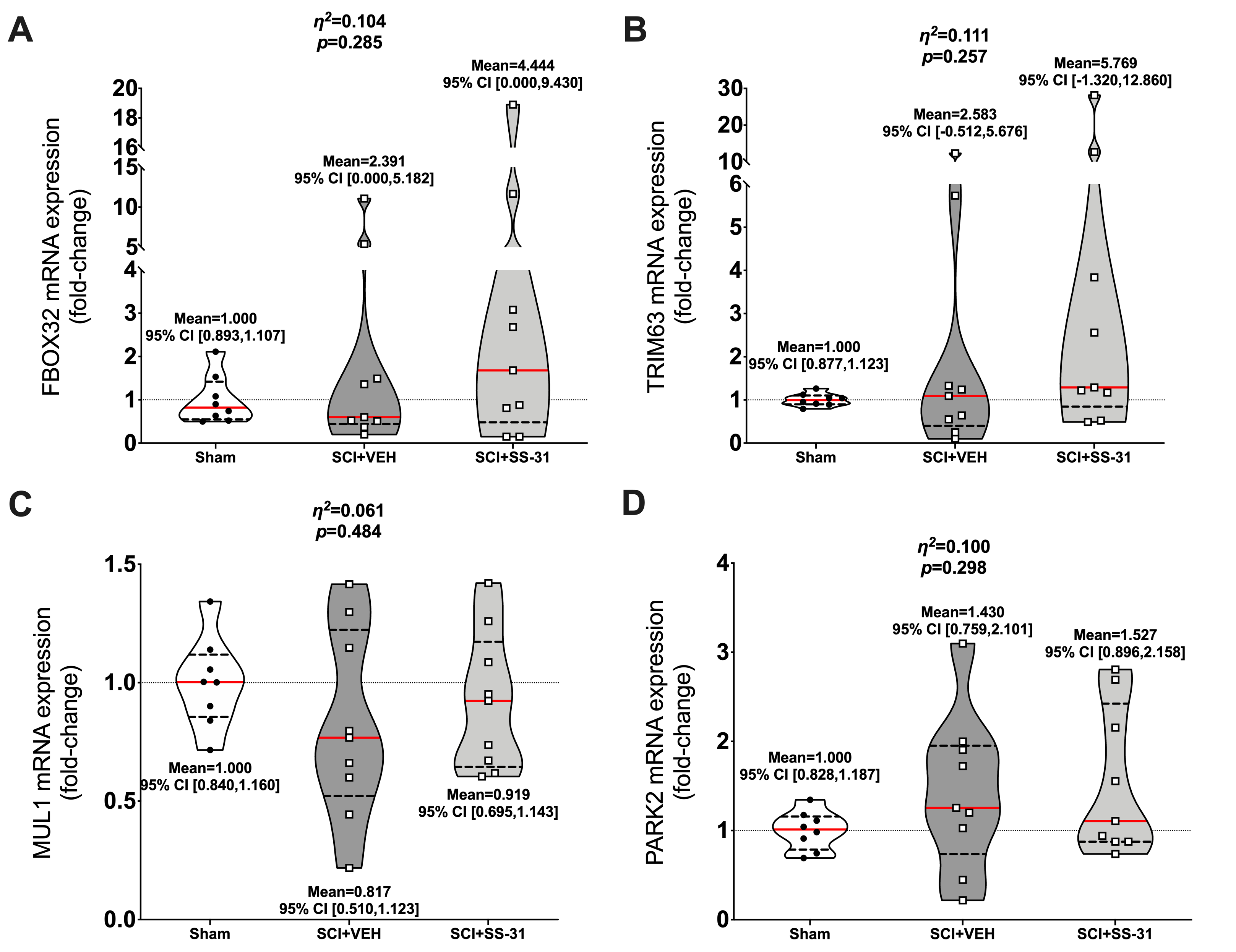

Supplement: Supplementary file 2 — Fig S2 [file PHY2-10-e15266-s003.tiff]
